# Investigating the Metabolic Benefits of Magnetic Mitohormesis in Patients with Type 2 Diabetes Mellitus

**DOI:** 10.3390/jcm14186413

**Published:** 2025-09-11

**Authors:** Fan Shuen Tseng, Gek Hsiang Lim, Yong Mong Bee, Phong Ching Lee, Yee Kit Tai, Alfredo Franco-Obregón, Hong Chang Tan

**Affiliations:** 1 Department of Endocrinology, Singapore General Hospital, Singapore 169856, Singapore; tseng.fan.shuen@mohh.com.sg (F.S.T.); bee.yong.mong@singhealth.com.sg (Y.M.B.); lee.phong.ching@singhealth.com.sg (P.C.L.); 2 Health Services Research Unit, Singapore General Hospital, Singapore 169856, Singapore; lim.gek.hsiang@sgh.com.sg; 3 Department of Surgery, Yong Loo Lin School of Medicine, National University of Singapore, Singapore 119228, Singapore; alextai@nus.edu.sg; 4 BICEPS Lab (Biolonic Currents Electromagnetic Pulsing Systems), National University of Singapore, Singapore 117599, Singapore; 5 Institute of Health Technology and Innovation (iHealthtech), National University of Singapore, Singapore 117599, Singapore

**Keywords:** diabetes mellitus, mitochondrial function, physical activity, endurance exercise, pulsed electromagnetic fields, magnetic mitohormesis, glycated hemoglobin, HbA1c, central obesity

## Abstract

**Background/Objectives**: Exercise is a key pillar in the management of type 2 diabetes mellitus (T2DM), but adherence rates to physical activity are poor. Pulsed electromagnetic field (PEMF) therapy, termed magnetic mitohormesis (MM), has been shown in preclinical and early human studies to mimic the metabolic benefits of exercise without physical strain. However, its effects on glycemic control remain unknown. We evaluate the metabolic benefits of MM in patients with suboptimally-controlled T2DM. **Methods**: An exploratory study was conducted in 40 adults with T2DM (glycated hemoglobin, HbA1c 7.0–10.0%). MM treatment comprised 12 sessions organized weekly, where low-dose PEMF was delivered to alternate legs for 10 min per visit. Metabolic assessments—anthropometry, HbA1c, fasting glucose and insulin resistance (measured by Homeostatic Model Assessment for Insulin Resistance, HOMA-IR)—were measured at baseline and post-treatment. Subgroup analysis was performed to compare the effects of MM on patients with and without central obesity (defined as waist-to-hip ratio ≥ 1.0). **Results**: Participants had a mean age of 59.4 years and HbA1c of 8.1%. MM treatment was well tolerated with no adverse events, and 77.5% of patients completed all 12 sessions. There were no significant changes in HbA1c, fasting glucose or HOMA-IR for the overall cohort. However, in patients with central obesity, 88.9% showed a reduction in HbA1c post-treatment compared to 32.3% without central obesity (*p* < 0.01), and mean HbA1c decreased from 7.5% to 7.1% (*p* < 0.01). **Conclusions**: Our findings suggest that MM is safe and well-tolerated in T2DM patients and may confer a preferential benefit for individuals with greater central obesity.

## 1. Introduction

Type 2 diabetes mellitus (T2DM) is a chronic metabolic disorder characterized by the human body’s inability to maintain normal blood glucose due to insulin resistance and relative insulin insufficiency [1]. Left uncontrolled, T2DM can lead to various micro- and macrovascular complications, which may be complicated by end-stage kidney disease, dialysis-dependence, blindness, and amputations. These complications lead to excess morbidity and mortality, lower the patient’s quality of life, and burden public health expenditure [2,3]. The prevalence of T2DM has been increasing worldwide and is projected to more than double from 529 million in 2021 to 1.31 billion in 2050 [2]. In Singapore, approximately 8.5% of Singapore residents suffer from diabetes, with rates of up to 24.2% amongst individuals aged 70 to 74 years [4]. An earlier projection estimated that the prevalence of T2DM could reach 15.0% by 2050 [5]. In response to this growing health concern, Singapore declared a war on diabetes in 2016 [6].

T2DM treatment requires pharmacological and lifestyle measures to maintain good blood glucose control. However, attaining glycemic control is challenging, with many patients unable to achieve optimal control for various reasons [7,8]. Patients with T2DM are commonly managed with multiple glucose-lowering therapies and medications, whereby compliance to treatment can be affected by high pill burden, financial barriers, and drug-associated adverse effects [9,10]. Nutritional lifestyle treatment for T2DM entails medical nutrition therapy and dietary weight loss, but is challenging to sustain [11,12]. Exercise also improves diabetes control and is strongly encouraged in T2DM patients [13,14,15,16].

Broadly, exercise can be divided into resistance and endurance forms of exercise. Whereas resistance exercise largely activates glycolytic (fast-twitch, type II) muscle fibers, endurance exercise engages the activity of mitochondria-rich oxidative (slow-twitch, type I) muscle fibers that are capable of sustained aerobic energy production from fatty acids [17]. As impaired mitochondrial function characterizes and contributes to the progression of T2DM [18], endurance exercise may help reverse T2DM-related mitochondrial dysfunction and improve glycemic control via the activation of the 5′ adenosine monophosphate-activated protein kinase (AMPK-) and peroxisome proliferator-activated receptor gamma coactivator 1-alpha (PGC-1α-) dependent pathways that are responsible for de novo mitochondriogenesis and increase skeletal muscle fatty acid oxidation [19,20]. Accordingly, endurance exercise has been shown to promote body weight reduction, improve cardiovascular risk profile and insulin sensitivity as well as increase glucose disposal by enhancing mitochondrial function [21,22]. Endurance exercise is thus recommended by the American Diabetes Association [14].

Despite the benefits of endurance exercise, rates of exercise and physical activity amongst patients with T2DM remain suboptimal, with poor adherence to the recommended 150 min or more per week of moderate-to-vigorous intensity physical activity [23,24,25]. The underlying factors are evident when considering the demographics of T2DM patients, many of whom are elderly and have co-existing medical conditions that present physical barriers to exercise or contribute to reduced exercise capacity (e.g., heart failure, visual impairment, poor bone health) [26].

We, and others, have shown that brief exposures to pulsed electromagnetic fields (PEMF) stimulate mitochondrial respiration via a calcium-mitochondrial axis upstream to PGC-1α transcriptional regulation and recreate biological and metabolic adaptations similar to endurance exercise but without physical stress or strain [27]. In pre-clinical murine studies, PEMF exposure was shown to activate muscle-mitochondrial respiration to induce exercise-related muscle adaptation and mitochondrial biogenesis. These responses resulted in the manifestation of typically exercise-associated positive metabolic adaptations, including improved insulin-sensitivity, reduced resting insulin levels, enhanced fatty acid oxidation, and enhanced oxidative muscle expression downstream of the well-established pro-metabolic health pathways largely governed by PGC-1α co-transcriptional regulation [28,29]. Related benefits have also been observed in several published human studies employing this same PEMF exposure paradigm. In elderly patients, brief 10-min weekly PEMF treatment for 12 weeks increased skeletal mass and reduced total and visceral adiposity [30]. More recently, it was found that PEMF treatment improved knee muscle strength and reduced pain in elderly patients with end-stage osteoarthritis of the knees [31]. In another example, weekly treatment with PEMF for 16 weeks improved markers of muscle mitochondrial functioning and lowered systemic lipotoxicity in patients who underwent anterior cruciate ligament reconstruction compared to placebo [32].

Collectively, these data support the ability of PEMF treatment to replicate the metabolic benefits of endurance exercise. However, it is unknown whether low-dose PEMF treatment, which we will refer to as magnetic mitohormesis (MM), improves diabetes control. In this open-labeled exploratory study, we investigated the impact of MM on metabolic control in patients with suboptimally-controlled T2DM. In addition, because PEMF treatment has been shown to reduce visceral fat [30], we will examine whether patients with central obesity (defined as waist-to-hip ratio, WHR of ≥1.0) exhibit a greater propensity to benefit more from this treatment.

## 2. Materials and Methods

### 2.1. Study Overview

We conducted a single-arm, exploratory study to investigate the impact of MM on patients with T2DM. This study was approved by the SingHealth Centralized Institutional Review Board (reference number: 2023/2044). Our trial was registered on ClinicalTrials.gov (identification number: NCT05881200).

### 2.2. Patient Recruitment

Adults with suboptimally-controlled T2DM were recruited from the Diabetes Centre in Singapore General Hospital from June 2023 to January 2024. Inclusion criteria were age 40–75 years, T2DM history of at least six months, glycated hemoglobin (HbA1c) between 7.0% to 10.0%, and body mass index (BMI) of 23.0 to 32.5 kg/m^2^. Exclusion criteria were patients with contraindications to PEMF exposure, medical contraindication to exercise, uncontrolled thyroid disease, systemic steroid usage, pregnancy, and uncontrolled hypertension. The full list of inclusion and exclusion criteria is available in Table 1. Written informed consent was obtained from all study participants before the initiation of study activities.

### 2.3. MM Treatment

**Figure 1:**
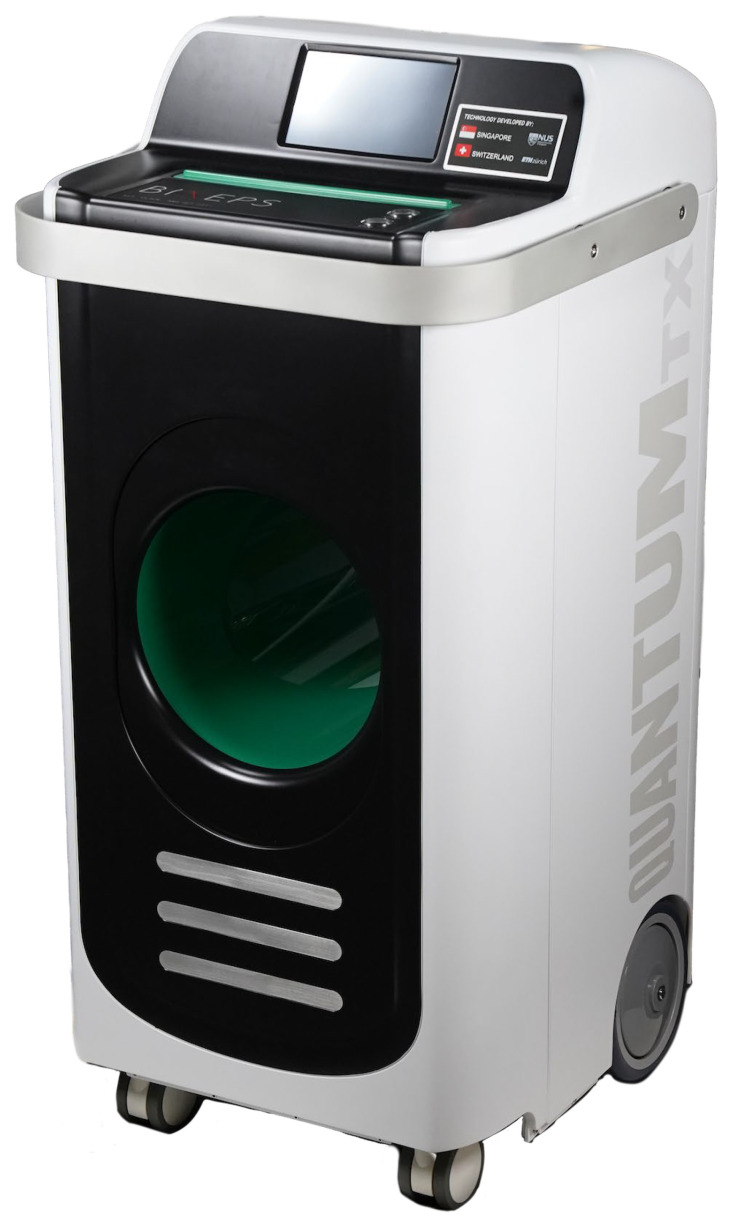
MM device used to deliver low-dose PEMF. The device measures 50 cm (W) × 60 cm (L) × 110 cm (H), with a treatment chamber with an internal diameter of 26 cm and a depth of 50 cm.

Low-dose PEMF was delivered using a commercial MM device (QuantumTX, Singapore) (Figure 1) as previously described [30,31]. The treatment protocol, including the frequency and duration of treatment, was consistent with our prior human studies [30,31,32,33]. It features a treatment chamber with an internal diameter of about 26 cm and a depth of 50 cm.

Magnetic field uniformity is of utmost importance in achieving the highest efficacy with the presented magnetic mitohormesis exposure paradigm [27,28]. The exposure system is based on a Helmholtz coil design wherein a limb placed anywhere within the lumen of the device is restricted to a region of highest field uniformity. The delivered fields are of sufficiently low frequency such that their delivery to muscles throughout the entire limb is not impeded. The device was designed to accommodate the leg musculature to ensure that a sufficiently large enough mass of muscle was exposed and stimulated by the low-energy fields to produce the largest amount of muscle secretome release into the bloodstream for systemic delivery [27].

For each MM session, participants sat on a standard chair and placed the target leg into the realm of the chamber. The device was aligned to ensure most of the upper thigh and quadriceps were situated within the chamber. Once in position, the device was activated to deliver a 10-min MM session at peak flux densities up to 1 mT. The subsequent session, scheduled for 4 to 9 days later, delivered PEMF to the alternate leg, and this continued until 12 sessions were completed. Subjects were required to maintain their baseline medications, dietary intake, and physical activities throughout the study duration.

### 2.4. Metabolic Assessments

**Figure 2:**
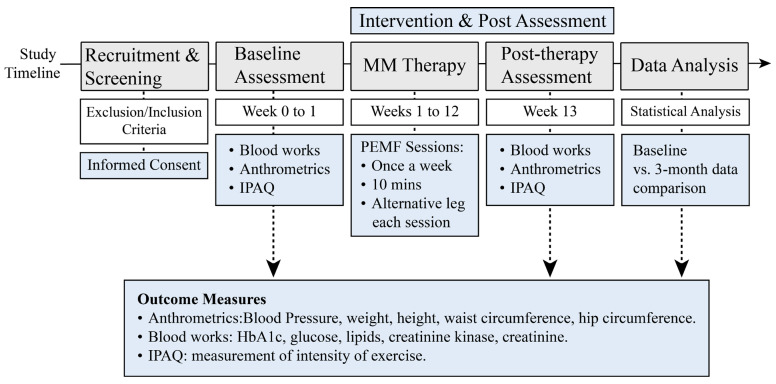
Study Timeline and Key Research Activities. This schematic outlines the design and flow of the study following screening and enrollment of eligible participants with T2DM.

Patients underwent a series of standardized metabolic assessments at baseline and after treatment at 3 months (Figure 2). Anthropometric measurements included blood pressure, weight, height, waist circumference, and hip circumference. Central obesity was defined as a waist-to-hip ratio of ≥1.0. Fasting blood samples were collected to measure HbA1c, glucose, lipids, creatinine kinase, and creatinine. Insulin resistance was estimated based on the Homeostatic Model Assessment for Insulin Resistance (HOMA-IR) [34]. The intensity of physical activity was measured using the short version of the International Physical Activity Questionnaire (IPAQ) [35]. Physical activities were reported as metabolic equivalents (METS) per week [36], and the participants were categorized as inactive, minimally active, and active.

### 2.5. Outcome Measures

The primary outcomes were the post-treatment reductions in fasting glucose, HbA1c, and insulin resistance (i.e., HOMA-IR).

### 2.6. Statistical Analysis

Data were first examined for normality. Normally distributed data were presented as mean ± standard deviation (SD), whereas non-normally distributed data were presented as median (interquartile range, IQR) or frequency (percentage). Normally distributed data measured at baseline and post-treatment within subjects were compared using the paired Student’s *t*-test, whereas corresponding non-normally distributed data were compared using the Wilcoxon signed-rank test. Between-group differences in categorical variables were tested using Pearson’s chi-squared test. Subgroup analyses were undertaken to examine whether MM treatment would preferentially benefit patients with central obesity. A two-tailed *p*-value of less than 0.05 was considered statistically significant. Statistical analyses were performed using Stata version 17 (StataCorp LLC, College Station, TX, USA) and Prism version 10 (GraphPad Software, Inc., Boston, MA, USA).

## 3. Results

**Figure 3:**
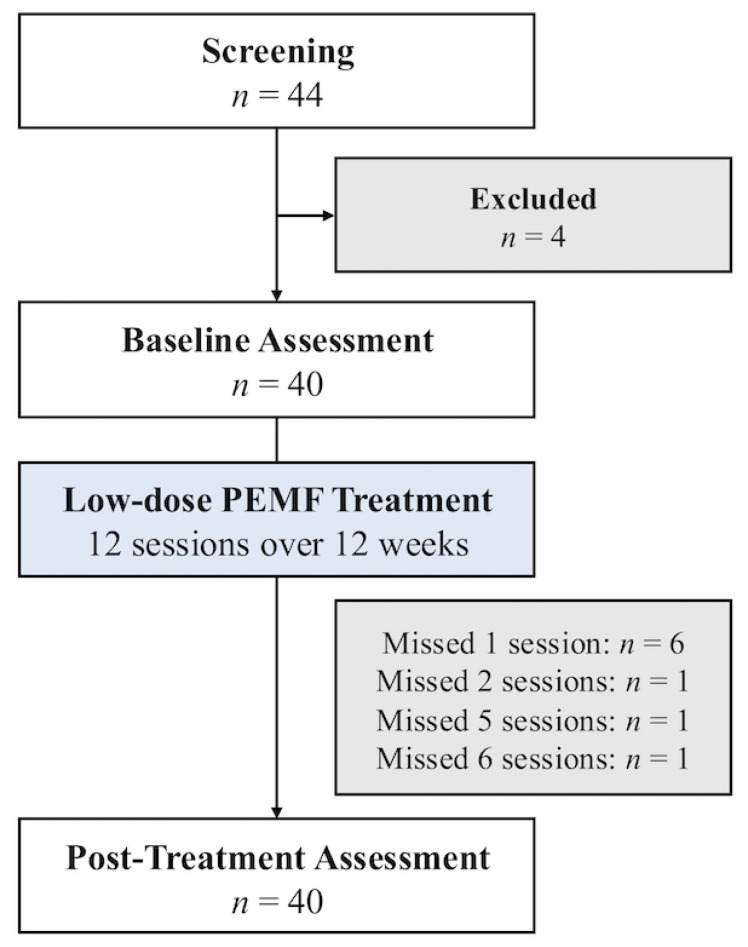
Study flow diagram of patients who underwent MM treatment.

Forty out of 44 subjects with T2DM were enrolled after screening for their eligibility (Figure 3). The baseline characteristics of the study subjects are summarized in Table 2. Patients had an average age of 59.4 ± 8.4 years and a duration of T2DM of 16.9 ± 9.4 years. The average baseline HbA1c was 8.1 ± 0.8%. The most used diabetes medications were metformin (95.0%), followed by sodium-glucose transport protein 2 (SGLT2) inhibitors (82.5%), sulfonylureas (47.5%), and dipeptidyl peptidase-4 (DPP-4) inhibitors (40.0%). There were no changes to diabetes medications throughout the study. 70.0% of participants had obesity, and 22.5% had central obesity. Their self-reported physical activity scores showed that 82.5% of the subjects were physically inactive and 15.0% were minimally active. Their post-treatment physical activity scores were also not significantly different from their baseline values. Common comorbidities were hyperlipidemia (87.5%) and hypertension (60.0%).

All subjects tolerated the MM well, and none dropped out of the study. Specifically, there were no significant adverse effects, such as musculoskeletal symptoms or increased serum creatinine kinase. 31 (77.5%) subjects completed all 12 sessions of MM therapy. Six (15.0%) subjects missed one session, and three (7.5%) subjects missed two or more sessions (Figure 3).

**Figure 4:**
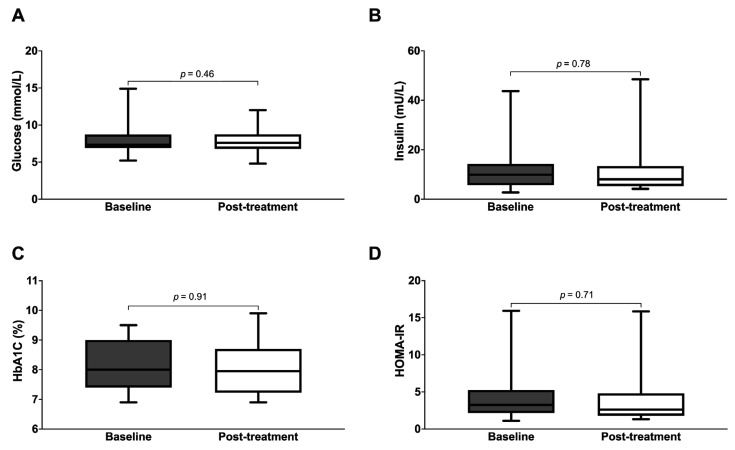
Metabolic parameters of subjects at baseline versus post-treatment ((**A**): fasting glucose; (**B**): insulin, (**C**): HbA1c, (**D**): HOMA-IR).

**Figure 5:**
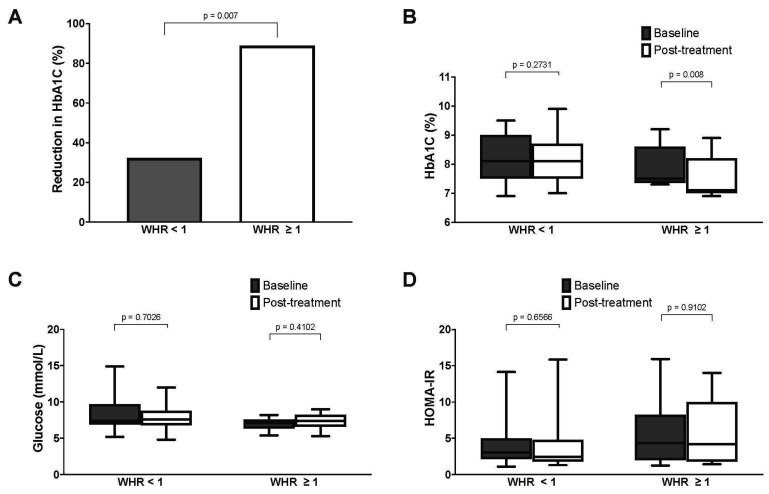
Comparison of metabolic parameters of subjects with and without central obesity (WHR of ≥1.0 and <1.0, respectively) ((**A**): percentage of subjects with reduction in HbA1c after treatment; (**B**): HbA1c; (**C**): fasting glucose; (**D**): HOMA-IR).

For the entire cohort, we did not observe any significant changes in glycemic control following treatment, as measured in the context of fasting glucose and HbA1c (Figure 4). Similarly, post-treatment fasting insulin and HOMA-IR were not significantly different from the baseline values. There were also no significant post-treatment changes to total weight, body composition, blood pressure, lipid profile, or renal function (Table 3). Similar results were obtained after excluding the nine subjects who missed one or more MM treatment sessions (Supplementary Table S1). However, subgroup analyses showed that a greater proportion of subjects with central obesity demonstrated a post-treatment reduction in HbA1c versus baseline, compared to those without central obesity (88.9% versus 32.3%, *p* < 0.01). Correspondingly, the HbA1c of subjects with central obesity decreased significantly from 7.5% (7.5–8.1) to 7.1% (7.0–8.9) (*p* < 0.01) (Table 4 and Figure 5). Subgroup analysis did not yield any significant post-treatment changes in serum fasting glucose, insulin, or HOMA-IR.

## 4. Discussion

To the best of our knowledge, we are the first to report on the therapeutic potential of MM to improve glycemic control in patients with suboptimal-controlled T2DM. In this single-arm study, we showed that MM therapy is safe in T2DM patients. In particular, T2DM patients with central obesity demonstrated a small, but statistically significant, improvement in HbA1c levels.

Similar to the subjects in our cohort, many patients with T2DM are diagnosed in middle age, struggle with multiple chronic conditions (including hyperlipidemia, hypertension and obesity), and require multiple glucose-lowering agents. The fact that 97.5% of patients in our cohort were inactive, or minimally active, is also a stark reflection of the low levels of physical activity amongst T2DM patients. The existing barriers to exercise are multifold and not easily overcome, comprising physiological, psychological, social, cultural, and environmental obstacles [37,38]. One of the greatest contributors to the ongoing diabetes epidemic is strongly linked to the rapidly aging population [39,40,41]. Clinicians must consider the practicality of frequent exercise in older patients who have concomitant frailty, sarcopenia, disability, and reduced physical function.

MM presents a safe and tolerable alternative to physical exercise that exerts minimal strain and stress on the body. Our study demonstrated a 100% retention rate, where most patients completed 10 min of 12 weekly sessions. Low-dose MM therapy is hence well-tolerated in T2DM patients, without any clinical or biochemical evidence of muscular injury. This is as opposed to conventional exercise, where overuse injuries such as strains or sprains are common amongst the older population [42,43,44].

**Figure 6:**
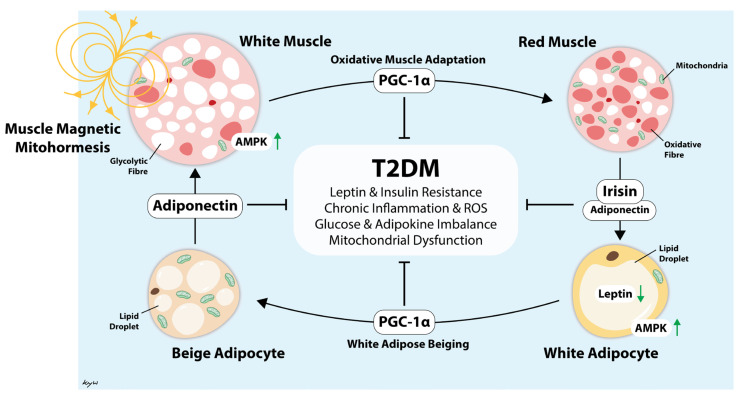
Illustration depicting how the muscle reddening-adipose browning cycle is instigated by muscle stimulation by magnetic mitohormesis exposure (or exercise) to offset systemic inflammation and metabolic dysfunction [17]. This chain of events is governed by the transcriptional co-regulator for mitochondriogenesis (PGC-1α) as previously cited [28,29]. Arrow heads are excitatory and blunted lines are inhibitory. Green arrows indicate increased or decreased protein activity or abundance.

Aerobic exercise has been shown in various meta-analyses to reduce HbA1c by a modest range of 0.3% to 0.7% [45,46,47,48,49,50]. Although the present study did not reveal any significant improvements in glycemic control, insulin resistance, cardiovascular markers, or anthropometry after three months of brief MM treatment, it did find that patients with central obesity exhibited statistically significant reductions in HbA1c, with a magnitude comparable to the benefits of aerobic exercise. WHR is an inexpensive and accessible anthropometric measurement that reflects visceral adiposity and central obesity [51,52]. This seemingly differential response to MM in patients with, and without, central obesity may be related to findings reported by Venugobal et al., who reported that PEMF increased lean muscle mass and reduced total body fat and visceral fat—all without significant changes in body weight [30]. Stephenson et al. also reported lower markers of ceramide lipotoxicity after PEMF [32]. We did not note any significant improvements in fasting glucose or HOMA-IR, possibly because these parameters primarily reflect hepatic insulin resistance and may not detect changes in skeletal muscle insulin resistance [53,54]. Hence, the observed improvements in HbA1c may be explained by the greater treatment effects of MM on skeletal muscle insulin resistance. Further studies are warranted to investigate the metabolic effects of MM in patients with T2DM, especially patients with central obesity. Evidence indicates that MM may lead to improvements in metabolic health through several mechanisms that synergize to ultimately reduce chronic inflammation via stimulated muscle-adipose paracrine crosstalk (Figure 6), resulting in adipose tissue beiging [17,29,55]. However, our pilot study aims to evaluate the potential clinical efficacy of MM in patients with suboptimal T2DM, and we did not perform any experiments that would provide a mechanistic explanation for the observation. Henceforth, future studies should include the measurement of pro-inflammatory peptides, cytokines, and immune cells.

Our data suggest that MM may be a safe alternative to conventional exercise. Its relatively brief, non-invasive and non-strenuous nature of implementation may serve to facilitate deployment and compliance in sedentary and frail clinical populations where exercise may prove prohibitive. Such treatment can be personalized to individual patients’ comorbidities and tolerance and could be especially effective in patients with specific metabolic phenotypes. However, as an exploratory single-arm study, our work has inherent limitations. The lack of a control group may fail to consider potentially degenerative trends observed in the normal disease course. The small sample size in this exploratory study limits the statistical power of the data. Controlled clinical trials would hence be crucial for establishing causal relationships and minimizing potential biases, allowing for more robust conclusions regarding the effectiveness of MM in T2DM. We have attempted to minimize potential confounders by ensuring patients maintained the same diet, baseline physical activity, and medications.

We believe that patients with central obesity, especially those with limited physical endurance or contraindications to aerobic exercise, would benefit the most from MM therapy. Central obesity is characteristic of severe metabolic disruption driven by mitochondrial impairment and inflammatory lipotoxicity. Visceral adiposity contributes to excessive production of ceramide species, a key contributor to the pathogenesis of T2DM [56]. We hypothesize that this group of patients has a higher baseline level of mitochondrial stress and ceramide-induced lipotoxicity, creating a greater potential for improvement with MM. As MM adaptively works via mild mitochondrial stress, it may counter this pathology by improving mitochondrial efficiency and redox signaling, resulting in significant reduction in HbA1c. This assumption is supported by preclinical studies indicating that muscle-targeted mitohormesis exerts strong adipogenic and anti-inflammatory effects [29,55] downstream of stimulated muscle-adipose paracrine crosstalk [57]. Human studies have similarly demonstrated magnetic mitohormetic-related changes in systemic lipotoxicity [32] and visceral adiposity [30] downstream of stimulated anti-inflammatory muscle-adipose paracrine crosstalk [33] that would ultimately serve to influence systemic lipid homeostasis (Figure 6). Nonetheless, before this treatment can be widely implemented, the optimal dose and duration of treatment that will produce the greatest clinical outcomes, such as reductions in micro- and macrovascular complications, must be determined. Furthermore, future studies should also examine whether combining MM with anti-diabetic medication of different classes and mechanisms of action would result in greater metabolic benefit. This is especially true with the newer classes of anti-diabetic drugs, specifically the SGLT2 inhibitors and GLP-1 agonists, which have been recently shown to significantly improve cardiorenal outcomes [58,59]. The potential of MM is supported by a recent study in healthy subjects, which found that twice-weekly MM treatment for one month produced significant changes in blood-borne myogenic and angiogenic biomarkers. This change translated into a sera with a significant capacity to stall breast cancer cell growth, migration, invasion, and transforming growth factor-beta (TGF-β)-dependent epithelial-mesenchymal transition [33]. Importantly, the anticancer potency of the sera was strongest one month after the MM treatment, indicating that the magnetic intervention had adapted muscles to become constitutive secretors of these factors.

While our study demonstrates clinical effects of MM on glycemic control, reflected as a reduction in HbA1c, the heterogeneity of T2DM necessitates a more nuanced investigation. To advance this field, future research should consist of large, phenotype-stratified randomized controlled trials (RCTs) and biomarker-driven RCTs designed around prominent T2DM phenotypes, such as insulin-resistant versus insulin-deficient profiles, or the presence of other significant comorbidities. These trials would benefit from incorporating deep metabolic phenotyping, including personalized lipidomic profiling (such as baseline ceramide levels and their modulation) to identify predictive biomarkers of clinical efficacy. A larger sample size, longer duration of MM treatment beyond 12 weeks, and/or more frequent MM treatment sessions per week may thus be needed to appreciate the metabolic benefits of the intervention and clarify for which patient profiles MM treatment is most effective.

While the current study should be interpreted within the context of its limitations, several key strengths merit emphasis. First, this investigation introduces a non-invasive therapeutic paradigm by targeting muscular mitochondria via MM in patients with T2DM. This approach is well-suited for patients who face challenges with conventional exercise or are contraindicated, such as those with severe obesity, orthopedic limitations, or cardiovascular comorbidities. Second, the intervention demonstrated feasibility and tolerability, evidenced by a 100% study completion rate and strong protocol adherence. The high compliance observed in this and other studies [31] is likely attributed to the passive, non-strenuous, and brief nature of the treatment, which requires minimal effort by the recipient. The level of acceptance is clinically significant and suggests MM is a practical modality for a diverse patient population. These strengths underscore the potential of MM as a unique and viable strategy for managing T2DM and provide a robust foundation for future research.

## 5. Conclusions

We report early data on the metabolic effects of MM treatment in patients with T2DM. In addition to having a good safety profile, low-dose PEMF shows promise in improving glycemic control, particularly in patients with central obesity. Larger, randomized controlled double-blinded trials are warranted to investigate the full extent of the impact of MM in improving clinical outcomes for patients with T2DM.

## Figures and Tables

**Table 1:** Full Inclusion and Exclusion Criteria of the Study.

Inclusion Criteria	Age 40–75 years T2DM history of at least six months HbA1c between 7.0 to 10.0% (most recent 3 months prior to enrolment) Body Mass Index (BMI) between 23.0 and 32.5 kg/m^2^ Able to ambulate independently Willing and able to give written informed consent
Exclusion Criteria	Presence of any conditions where PEMF exposure is contraindicated (e.g., active electronic implants, cardiac implantable electronic devices) Medical advice against physical activity Chest pain at rest or when performing physical activity Blood pressure > 180/90 mmHg Women who are pregnant, considering pregnancy or lactating Cancer not in remission, or receiving active cancer treatment Current participation in another clinical trial Systemic steroid usage Uncontrolled thyroid disease Significant alcohol intake (>1 unit per day for women and >2 units per day for men) Any factors likely to limit adherence to study protocol (e.g., dementia; alcohol or substance abuse; history of unreliability in medication taking or appointment keeping; significant concerns about participation in the study from spouse, significant other or family members) Anticipated surgery or changes in diabetes medications during the study duration History of severe hypoglycemia in the recent three months Previous usage of any MM device in the past three months Recent surgical procedure in the last six months, where muscle activation can interfere with the healing response

**Table 2:** Baseline characteristics of participants.

	Subjects (*n* = 40)
Age (years), mean ± SD	59.4 ± 8.4
Male, *n* (%)	18 (45.0)
Ethnicity, *n* (%)	
Chinese	28 (70.0)
Malay	4 (10.0)
Indian	6 (15.0)
Others	2 (5.0)
Obesity, *n* (%)	28 (70.0)
Central obesity (waist-to-hip ratio ≥ 1.0), *n* (%)	9 (22.5)
Regular alcohol intake, *n* (%)	3 (7.5)
Duration of DM (years), mean ± SD	16.9 ± 9.4
Comorbidities, *n* (%)	
Hypertension	24 (60.0)
Hyperlipidemia	35 (87.5)
Heart failure	1 (2.5)
Stroke	0 (0.0)
On diabetes medications, *n* (%)	39 (97.5)
Types of medications, *n* (%)	
Sulfonylureas	19 (47.5)
SGLT2 inhibitors	33 (82.5)
Metformin	38 (95.0)
DPP-4 inhibitors	16 (40.0)
Thiazolidinediones	2 (5.0)
GLP-1 receptor agonists	6 (15.0)
Insulin	9 (22.5)
Total physical activity (METS minutes per week), median (IQR)	1308 (753–2079)
Level of physical activity, *n* (%)	
Inactive	33 (82.5)
Minimally active	6 (15.0)
Active	1 (2.5)

**Table 3:** Clinical and laboratory parameters of participants at baseline versus post-MM treatment.

	Baseline (*n* = 40)	Post-MM (*n* = 40)	*p*-Value
Waist-to-hip ratio, median (IQR)	0.95 (0.93–0.99)	0.96 (0.92–1.00)	>0.99
Waist circumference (cm), median (IQR)	98.0 (91.5–101.8)	97.3 (91.5–103.3)	0.72
Hip circumference (cm), mean ± SD	100.9 ± 5.9	101.0 ± 5.8	0.89
Weight (kg), median (IQR)	69.7 (63.2–75.9)	70.4 (62.5–76.0)	0.97
Body mass index (kg/m^2^), mean ± SD	26.6 ± 2.4	26.5 ± 2.4	0.54
Fat mass (kg), median (IQR)	23.1 (18.8–28.0)	22.7 (17.9–25.3)	0.78
Fat-free mass (kg), mean ± SD	47.5 ± 9.6	47.1 ± 9.9	0.31
Systolic BP (mmHg), median (IQR)	123.0 (116.0–132.0)	122.0 (114.0–133.0)	0.89
Diastolic BP (mmHg), median (IQR)	73.0 (65.0–78.5)	71.0 (65.0–78.0)	0.70
HbA1c (%), mean ± SD	8.1 ± 0.8	8.0 ± 0.8	0.67
HOMA-IR, median (IQR)	3.2 (2.2–5.2)	2.6 (1.8–4.8)	0.70
Insulin (mU/L), median (IQR)	9.9 (5.7–14.3)	8.1 (5.3–13.4)	0.82
Fasting glucose (mmol/L), median (IQR)	7.4 (6.9–8.6)	7.6 (6.8–8.7)	0.45
Creatinine (µmol/L), median (IQR)	68.0 (52.0–87.5)	67.5 (54.0–88.5)	0.82
eGFR (ml/min), median (IQR)	97.5 (74.5–107.0)	99.0 (81.0–107.0)	0.38
Creatinine Kinase (U/L), median (IQR)	88.5 (66.5–147.0)	85.5 (65.0–119.5)	0.40
Total cholesterol (mmol/L), median (IQR)	4.0 (3.6–4.7)	4.0 (3.7–4.9)	0.94
HDL-cholesterol (mmol/L), mean ± SD	1.2 ± 0.3	1.3 ± 0.3	0.37
LDL-cholesterol (mmol/L), median (IQR)	2.1 (1.6–2.5)	2.2 (1.8–2.7)	0.87
Triglycerides (mmol/L), median (IQR)	1.2 (1.0–1.6)	1.3 (1.0–1.6)	0.62
Total physical activity (METS minutes per week), median (IQR)	1308 (753–2079)	1181 (594–1964)	0.50

Normally distributed variables were compared with paired Student’s *t*-test, whereas Wilcoxon’s signed-rank test was used for non-normally distributed variables.

**Table 4:** Differential response to MM in patients with and without central obesity (waist-to-hip ratio, WHR of ≥1.0 and <1.0, respectively).

	WHR ≥ 1.0 (with Central Obesity) (*n* = 9)			WHR < 1.0 (Without Central Obesity) (*n* = 31)		
	Baseline	Post-MM	*p*-Value	Baseline	Post-Treatment	*p*-Value
Reduction in HbA1c, *n* (%)		8 (88.9)			10 (32.3)	<0.01
HbA1c (%), median (IQR)	7.5 (7.5–8.1)	7.1 (7.0–8.9)	<0.01	8.1 (7.5–9.0)	8.1 (7.5–8.7)	0.97
Insulin (mU/L), median (IQR)	14.2 (7.8–18.5)	13.3 (6.0–18.5)	0.68	8.8 (5.6–12.4)	7.9 (5.3–12.8)	0.57
Fasting glucose (mmol/L), median (IQR)	7.1 (6.0–7.4)	7.4 (6.7–7.9)	0.41	7.4 (6.9–9.7)	7.6 (6.8–8.8)	0.70
HOMA-IR, median (IQR)	4.3 (2.5–5.8)	4.2 (1.8–6.5)	0.91	3.0 (2.1–5.0)	2.4 (1.8–4.8)	0.66

Non-normally distributed were compared with Wilcoxon’s signed-rank test.

## Data Availability

All data generated in this study are presented in the article. The raw 896 data supporting the findings are available from the corresponding author upon request.

## Supplementary Materials

The following supporting information can be downloaded at: https://www.mdpi.com/article/10.3390/jcm14186413/s1, Table S1: Clinical and laboratory parameters of participants at baseline versus post-MM treatment for subjects who completed all 12 MM sessions (i.e., excluded subjects who missed one or more MM sessions).

## Abbreviations

The following abbreviations are used in this manuscript:

BP	blood pressure
DM	diabetes mellitus
DPP-4	dipeptidyl peptidase-4
eGFR	estimated glomerular filtration rate
HbA1c	glycated hemoglobin
HDL-C	high-density lipoprotein cholesterol
HOMA-IR	Homeostatic Model Assessment for Insulin Resistance
LDL-C	low-density lipoprotein cholesterol
METS	metabolic equivalent task
MM	magnetic mitohormesis
PEMF	pulsed electromagnetic field
SGLT2	sodium-glucose transport protein 2
T2DM	type 2 diabetes mellitus
WHR	waist-to-hip ratio
