# Supplementary material for: Investigating the Metabolic Benefits of Magnetic Mitohormesis in Patients with Type 2 Diabetes Mellitus
Source: J Clin Med. 2025 Sep 11;14(18):6413. doi: 10.3390/jcm14186413 (PMC12470278; doi:10.3390/jcm14186413)
Supplement: Supplementary file 1 [file jcm-14-06413-s001.zip › jcm-3831765-supplementary.pdf]

Supplementary Table 1 Clinical and laboratory parameters of participants at baseline versus post-MM treatment for subjects who completed all twelve MM sessions (i.e., excluded subjects who missed one or more MM sessions)

	**Baseline (n = 31)**	**Post-treatment (n = 31)**	**P value**
Waist-to-hip circumference, cm	0.95 (0.93-0.99)	0.96 (0.92-1.00)	0.76
Waist circumference, cm	99.0 (93.0-102.5)	98.0 (92.0-103.0)	0.83
Hip circumference, cm	102.0 ± 5.8	101.9 ± 5.7	0.83
Weight, kg	70.0 (65.1-75.4)	70.5 (60.5-75.4)	0.97
Body mass index, kg/m2	27.0 ± 2.3	26.9 ± 2.4	0.24
Fat mass, kg	24.3 (19.4-28.8)	24.2 (18.1-28.5)	0.82
Fat free mass, kg	47.5 ± 9.4	46.8 ± 9.7	0.26
SBP, mmHg	125 (116-134)	121 (113-128)	0.68
DBP, mmHg	73 (68-79)	71 (65-78)	0.49
HbA1C, %	8.0 ± 0.8	8.0 ± 0.9	0.65
HOMA-IR	3.0 (1.9-4.4)	2.4 (1.8-4.3)	0.54
Insulin, mU/L	8.8 (5.1-13.7)	7.9 (5.3-12.8)	0.97
Glucose, mmol/L	7.3 (7.0-8.9)	7.6 (6.8-8.6)	0.84
Creatinine, umol/L	63.0 (52.0-81.0)	61.0 (53.0-78.0)	0.67
GFR, ml/min	100.0 (85.0-107.0)	100.0 (95.0-109.0)	0.12
Creatinine Kinase, U/L	90.0 (68.0-137.0)	85.0 (65.0-118.0)	0.38
Total cholesterol, mmol/L	4.1 (3.5-4.7)	4.0 (3.5-4.8)	0.46
HDL, mmol/L	1.2 ± 0.3	1.2 ± 0.3	0.86
LDL, mmol/L	2.1 (1.6-2.7)	2.1 (1.7-2.7)	0.57
Triglycerides, mmol/L	1.2 (1.0-1.5)	1.2 (0.9-1.6)	0.46
Total physical activity (METS min/week)	1091 (693-1848)	1230 (495-2079)	0.43

Normally distributed variables were compared with paired Student’s t-test, whereas Wilcoxon’s signed-rank test was used for non-normally distributed variables. BP, blood pressure; eGFR, estimated glomerular filtration rate; HbA1c, glycated hemoglobin; HDL, high-density lipoprotein; HOMA-IR, Homeostatic Model Assessment for Insulin Resistance; LDL, low-density lipoprotein; METS, metabolic equivalent task.
